# Analysis of molecular mechanisms of delafloxacin resistance in *Escherichia coli*

**DOI:** 10.1038/s41598-024-78124-9

**Published:** 2024-11-02

**Authors:** András Kubicskó, Katalin Kamotsay, Dóra Szabó, Béla Kocsis

**Affiliations:** 1 Institute of Medical Microbiology, Semmelweis University, 1089 Budapest, Hungary; 2 Central Microbiology Laboratory, National Institute of Hematology and Infectious Disease, Central Hospital of Southern-Pest, 1097 Budapest, Hungary; 3 HUN-REN-SU Human Microbiota Research Group, 1052 Budapest, Hungary; 4 Department of Neurosurgery and Neurointervention, Semmelweis University, 1083 Budapest, Hungary

**Keywords:** Delafloxacin, Fluoroquinolone resistance, WGS, ESBL, AmpC, High-risk clone, Microbiology, Diseases, Infectious diseases

## Abstract

In this study delafloxacin resistance mechanisms in *Escherichia coli* strains were analyzed. Delafloxacin is a new fluoroquinolone, that is approved for clinical application however, resistance against this agent is scarcely reported. In our study 37 *E. coli* strains were included and antimicrobial susceptibility testing was performed for ciprofloxacin, delafloxacin, levofloxacin, moxifloxacin, ceftazidime, cefotaxime, imipenem. Six delafloxacin resistant *E. coli* strains were selected for whole-genome sequencing and all of them exhibited resistance to other fluoroquinonlones and showed an extended-spectrum beta-lactamase phenotype. The six delafloxacin resistant *E. coli* strains belonged to different sequence types (STs) namely, ST131 (2 strains), ST57 (2 strains), ST162 and ST15840. Each delafloxacin resistant strain possessed multiple mutations in quinolone resistance-determining regions (QRDRs). Notably, three mutations in *gyrA* Ser83Leu, Asp87Asn and *parC* Ser80Ile were in strains of ST162, ST57 and ST15840. However, the two strains of ST131 carried five combined mutations namely, *gyrA* Ser83Leu, Asp87Asn, *parC* Ser80Ile, Glu84Val, *parE* Ile549Leu. Association of delafloxacin resistance and production of CTX-M-15 in ST131, CMY-2 in ST162 and ST15840 was detected. In this study a new ST, ST15840 of clonal complex 69 was identified. Our results demonstrate, that at least three mutations in QRDRs are required for delafloxacin resistance in *E. coli*.

## Introduction

*Escherichia coli* is a facultative anaerobe bacterium and a common inhabitant of human gastrointestinal tract. However, pathogenic *E. coli* strains acquire virulence factors, that enable them to cause a variety of diseases. *E. coli* has been classified into different pathotypes, that are causative agents of intestinal infections. Beyond gastrointestinal infections, *E. coli* has the ability to cause diverse extraintestinal diseases, including urinary tract infection, peritonitis, bacteriaemia and meningitis^1–5^.

Antimicrobial resistance in *E. coli* has been described worldwide^1,6,7^. Multidrug-resistant *E. coli* clones have disseminated and these are great challenges in hospital wards, because antibiotic resistant *E. coli* causes high-number of difficult-to-treat infections, that are associated with high mortality rates and with different complications^8^. Among antibiotic resistance mechanisms in *E. coli*, beta-lactam resistance is confered by production of different beta-lactamases namely, extended-spectrum beta-lactamases (ESBLs) (e.g.: SHV, TEM, CTX-M), AmpC type β-lactamases (e.g.: ACC, CMY, DHA) and OXA-type beta-lactamases cause resistance to penicillins and cefalosporins. However, carbapenem resistance in *E. coli* is mainy conferred by metallo-β-lactamases (e.g.: VIM, NDM, IMP), *Klebsiella pneumoniae* carbapenemase (KPC) and OXA-48-like beta-lactamases^9–14^.

Resistance to fluoroquinolones in *E. coli* is explained by the accumulation of mutations in quinolone resistance-determining regions (QRDRs), that are located in coding genes of gyrase (*gyrA*, *gyrB*) and topoisomerase IV (*parC*, *parE*)^15,16^. Plasmid-mediated quinolone resistance (PMQR) determinants were described in *E. coli* and in other members of Enterobacterales, that enhance development of fluoroquinolone resistance through the increase in mutation frequency^17^. PMQR includes Qnr determinants (QnrA, QnrB, QnrC, QnrD, QnrE), Aminoglycoside-acetyltransferase-(6’)-Ib-cr [*aac(6’)-Ib-cr*] variant as well as QepA and OqxAB efflux pumps^18–21^. PMQRs and beta-lactamase genes are located usually jointly on plasmids, that can be transferred between different species of Enterobacterales furthermore, these resistance plasmids can harbour additional resistance markers, that confer resistance to aminoglycosides, fosfomycin and colistin^22–24^.

Delafloxacin is a new fluoroquinolone agent, that was approved for clinical applications in the past years^25–28^. Delafloxacin has a broad antibacterial spectrum, that includes a wide range of bacteria: gram-positive cocci (staphylococci, including methicillin-resistant *Staphylococcus aureus* (MRSA) and methicillin-sensitive *S. aureus* (MSSA), streptococci, and enterococci), gram-negatives (e.g.: *Pseudomonas aeruginosa*, *Acinetobacter baumannii*, *Haemophilus influenzae*, *Moraxella catarrhalis*, ESBL-producing *E. coli* and *K. pneumoniae*, *Neisseria gonorrhoeae*, and *Helicobacter pylori*), anaerobes (e.g.: *Bacteroides fragilis*), and causative agents of atypical pneumonia, including *Legionella pneumophila*, *Chlamydia pneumoniae*, and *Mycoplasma pneumoniae*^29–31^. Delafloxacin targets both bacterial DNA gyrase and topoisomerase IV enzymes of gram-positive and gram-negative bacteria with equal affinity. Delafloxacin is a bactericidal agent and it has a larger molecular surface compared to earlier fluoroquinolones, that enables it with more binding sites on the target molecules. Delafloxacin has an enhanced antibacterial efficacy in acidic environments, compared to previous fluoroquinolones, and additionally it inhibits biofilm formation in *S. aureus*^32^. Delafloxacin has been approved to treat acute bacterial skin and skin-structure infections, and community-acquired bacterial pneumonia, and it has a potential indication in therapy of intra-abdominal infections and in complicated urinary tract infection^28,33^.

The aim of this study was to analyze delafloxacin resistance mechanisms in *E. coli* strains.

## Materials and methods

This study was approved by Ethics Committee of Semmelweis University (SE RKEB: 218/2020). All procedures of this study were in accordance with the ethical standards of the Institutional National Research Committee and with the 1964 Declaration of Helsinki and its later amendments or comparable ethical standards. Participants of this study gave written informed consent for sample collection and analysis.

### Strains

Altogether 90 non-repetitive *E. coli* strains were collected between September and December 2022 at South-Pest Central Hospital, National Institute of Hematology and Infectious Diseases, Budapest, Hungary, from various clinical samples, including hemoculture and urine. All isolates were identified by matrix-assisted laser desorption ionization time-of-flight mass spectrometry (MALDI Biotyper, Bruker, Bremen, Germany). The inclusion criteria of *E. coli* strains in this study were resistance to ciprofloxacin and/or resistance to third-generation cephalosporins or a confirmed extended-spectrum beta-lactamase (ESBL) positivity by double-disk synergy test. Upon these inclusion criteria 37 *E. coli* strains were selected for the antibiotic susceptibility testing of this study.

## Determination of the minimum inhibitory concentration (MIC)

Antibiotic susceptibility testing was performed for delafloxacin, ciprofloxacin, levofloxacin, moxifloxacin, ceftazidime, cefotaxime, and imipenem. MIC values were determined by broth microdilution method in Muller–Hinton broth in 96-well microplates.

The MIC results were interpreted according to the latest EUCAST protocol v14.0 (http://www.eucast.org/), accessed on 1 January 2024. *E. coli* ATCC 25922 was the control strain in this study. Delafloxacin was purchased from Sigma-Aldrich GmbH, Schnelldorf, Germany; Levofloxacin was purchased from TEVA Zrt Debrecen, Hungary; Moxifloxaxin was purchased from Bayer Schering Pharma AG, Berlin, Germany; Ciprofloxacin was purchased from Fresenius Kabi Bad Homburg Germany; Imipenem was purchased from Fresenius Kabi Budapest, Hungary; Cefotaxim was purchased from Sanofi-Aventis Zrt, Budpest Hungary; ceftriaxon was purchased from Fresenius Kabi Bad Homburg Germany.

## Whole-genome sequencing (WGS)

WGS analysis was performed on six selected *E. coli* strains (SE-LKH *E. coli* 35, *E. coli* 133, *E. coli* 149/1, *E. coli* 260, *E. coli* 269/1, *E. coli* 706/1). The selection criteria for WGS were *E. coli* strains exhibiting ciprofloxacin and delafloxacin resistance as well as ESBL phenotype. WGS was performed by Illumina MiSeq system in Eurofins BIOMI Kft (Gödöllő, Hungary) as reported earlier^34^. Briefly, genomic DNA was extracted by NucleoSpin Microbial DNA Mini kit (Macherey-Nagel, Düren, Germany). Amount of isolated DNA was measured by Qubit fluorometer, and quality of DNA was tested by microcapillary electrophoresis (Tape Station 4150, Agilent, Waldbronn, Germany). Libraries were prepared by Illumina DNA Prep kit, according to the manufacturer’s instruction. Sequencing was performed on an Illumina Miseq system using MiSeq Reagent Kit v2 generating 250 bp paired-end reads. Genome assembly was performed with the SPAdes Genome assembler algorithm v3.15.3. Antibiotic-resistance genes were detected in the assembled genomes by Bionumerics v8.1 software^34^. Assembled genomes of *E. coli* strains were analyzed by the traditional 7 gene multilocus sequence typing (MLST). Whole genome MLST (wgMLST) was performed based on 17,350 gene allel variants by Bionumerics v8.1.

## Results

### Antimicrobial susceptibility testing

**Fig. 1:**
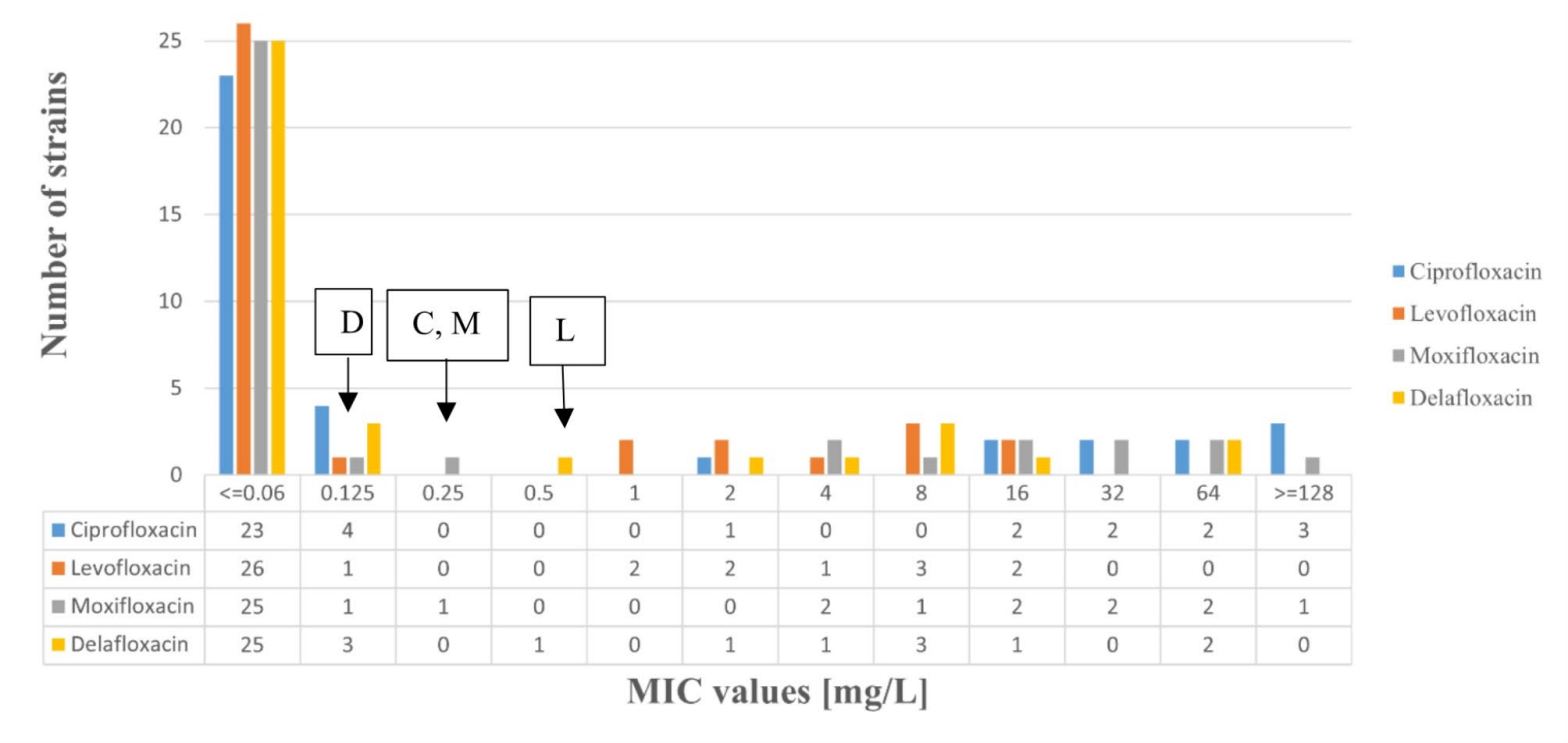
Fluoroquinolone MIC distribution in 37 *E. coli* strains of this study. Breakpoints of ciprofloxacin (C), delafloxacin (D), levofloxacin (L), and moxifloxacin (M) are shown.

In our study 37 *E. coli* clinical isolates were included and fluoroquinolone MIC distribution of strains is shown on Fig. 1. Among our strains in this study 27 were susceptible to ciprofloxacin, levofloxacin and moxifloxacin however, 28 were susceptible to delafloxacin. Altogether 9 *E. coli* strains exhibited resistance to delafloxacin and these strains were resistant to other tested fluoroquinolones.

Association of fluoroquinolone resistance and ESBL phenotype was detected in our collection. Notably, 26% of *E. coli* strains in this study exhibited fluoroquinolone resistance and showed ESBL phenotype however, 32% of strains were fluoroquinolone resistant and lacked ESBL phenotype, while 42% of strains exhibited ESBL phenotype but remained susceptible to fluoroquinolone. All *E. coli* strains were susceptible to imipenem in this study.

### Genome sequencing

The six delafloxacin resistant *E. coli* belonged to different sequence types (STs) according to PubMLST (Achtman) results namely, ST131 (two strains), ST57 (two strains), ST162 (one strain) and ST15840 (one strain) (Table 1). *E. coli* 133 of our study belongs to the new ST, ST15840, that is closely related to ST69, and a member of clonal complex 69. According to allelic variations, the *icd* gene of ST15840 differed in a single mutation (C426T) from *icd* wild-type gene, that indicated the novel 1994 allel and determined a new ST namely, ST15840. Genomic data of ST15840 are submitted to Enterobase database at SE-LKH-*E. coli* 133 name.

**Fig. 2:**
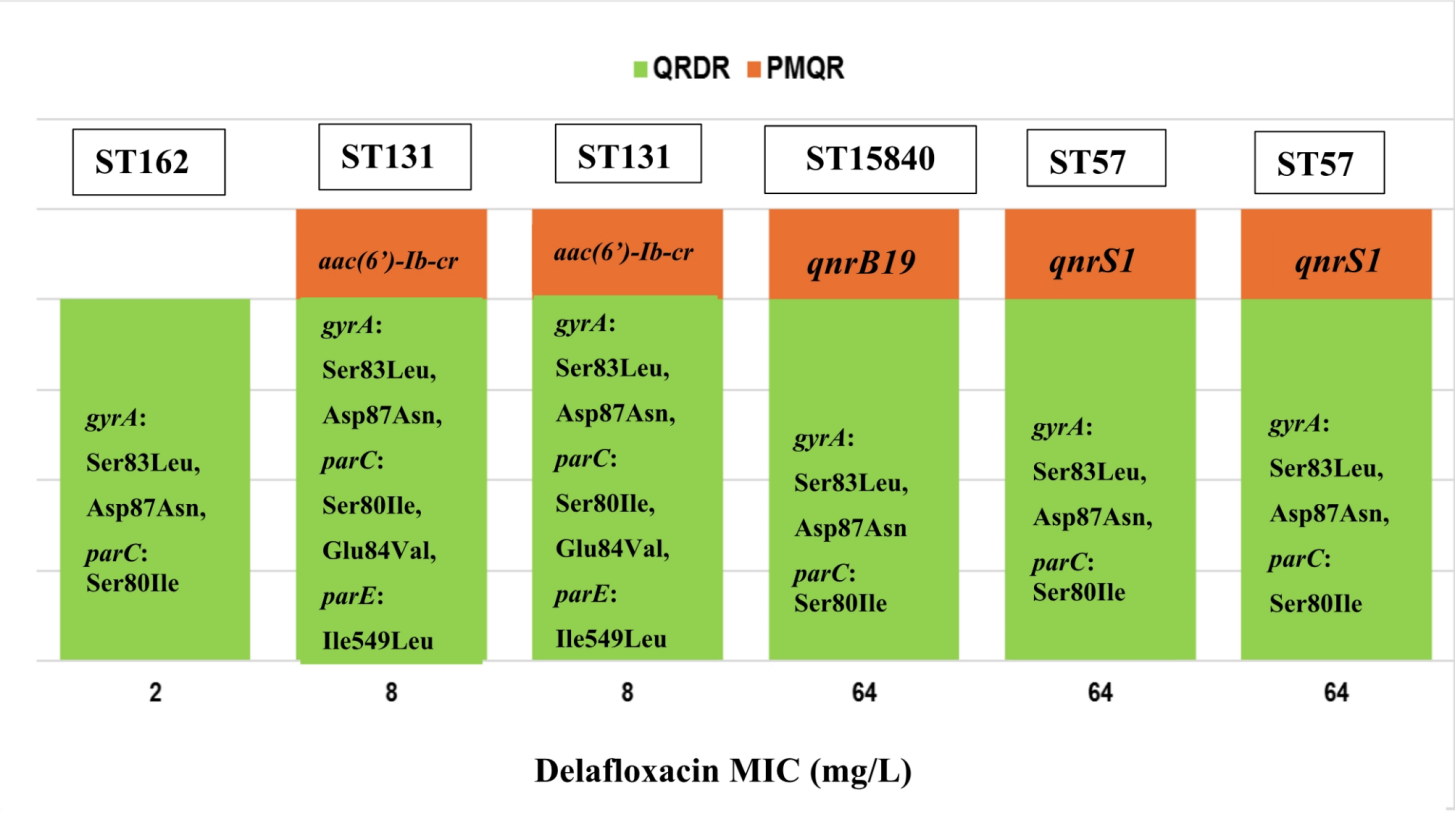
QRDR mutations and PMQR determinants in delafloxacin resistant *E. coli* strains in correlation with delafloxacin MIC values. Three mutations of QRDRs are sufficient for delafloxacin resistance. Among PMQR determinants *aac(6’)-Ib-cr*,* qnrB19* and *qnrS1* can have additional role in increment of delafloxacin MIC values. (QRDR = quinolone resistance-determining region; PMQR = plasmid-mediated quinolone resistance).

WGS analyis of six delafloxacin resistant *E. coli* strains proved multiple mutations in QRDRs, as well as presence of different PMQR determinants. Triple mutations in QRDRs namely, *gyrA* Ser83Leu, Asp87Asn, and *parC* Ser80Ile were detected in strains of ST162, ST57 and ST15840. However, two strains of ST131 carried five combined mutations namely, *gyrA* Ser83Leu, Asp87Asn, *parC* Ser80Ile, Glu84Val, *parE* Ile549Leu (Fig. 2).

**Fig. 3:**
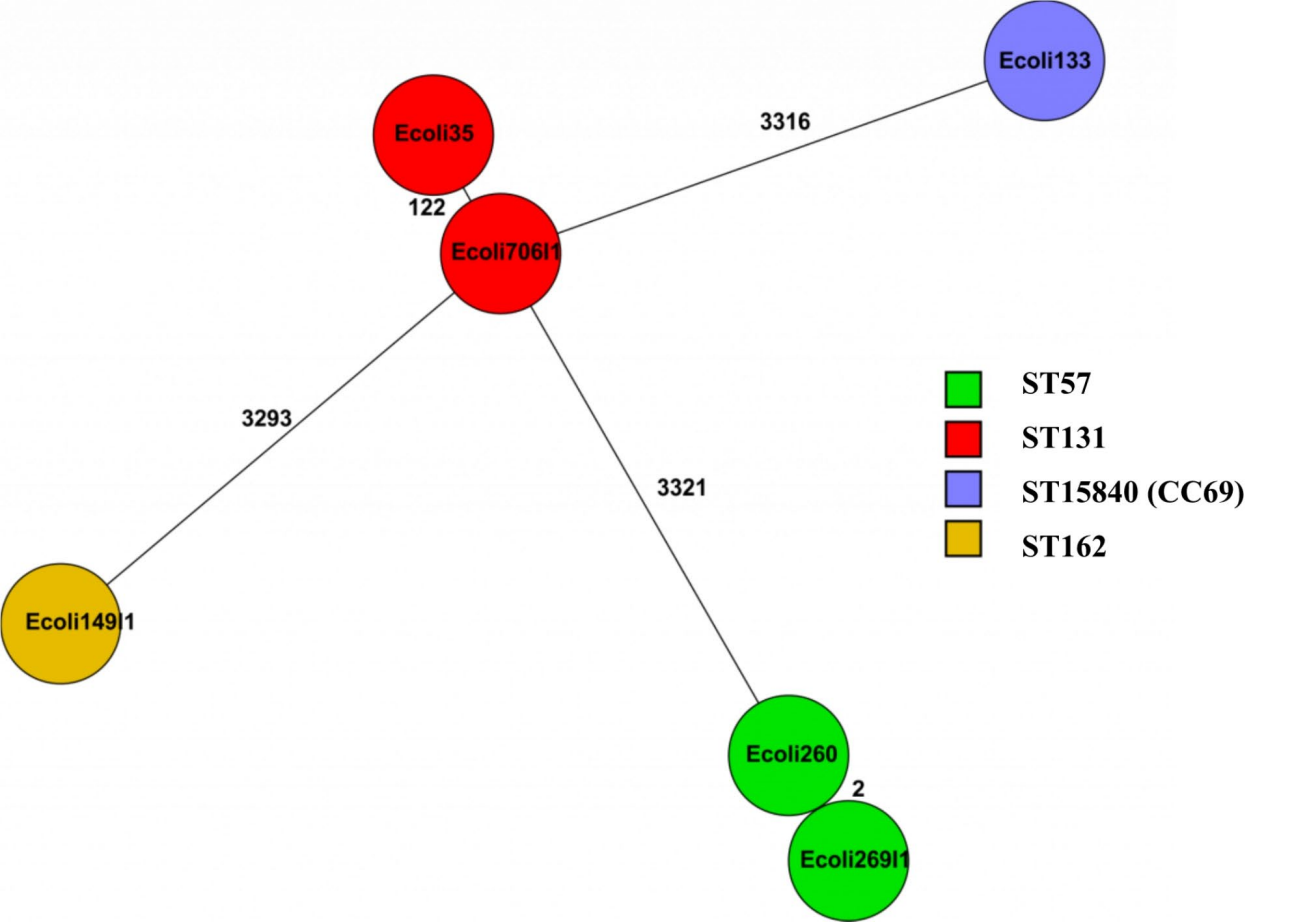
Minimum spanning tree based on whole genome MLST analysis of six delafloxacin resistant *E. coli* strains. This figure illustrates ST131 (2 strains), ST57 (2 strains), ST162, and ST15840 (clonal complex 69). Each circle represents a strain. Colour of circle indicates sequence type (ST) of strain. Connecting lines between circles show number of allelic differences between the two strains.

PMQRs were also detected in delafloxacin resistant *E. coli* strains namely, *qnrS1* in strains of ST57, *qnrB19* in strain of ST15840, and *aac(6’)-Ib-cr* in strains of ST131 (Fig. 2). Additionally, other resistance determinants, *sul1*, *sul2*, *dfrA17*, *aac(3)-IIa*, *aac(3)-IId*, were also detected in *E. coli* strains of this study. Different beta-lactamases were associated with delafloxacin resistant *E. coli* strains namely, CTX-M-15, CMY-2, OXA-1 and TEM-1B (Table 1). The wgMLST analysis of six delafloxacin resistant *E. coli* strains is shown on Fig. 3.

**Table 1:** Six delafloxacin resistant *E. coli* strains are demonstrated. Antibiotic resistance genes, plasmid-mediated quinolone resistance (PMQR) determinants, multilocus sequence type (MLST), MIC values (mg/L) for ciprofloxacin (cip), levofloxacin (lev), moxifloxacin (mox), delafloxacin (del), ceftazidime (caz), cefotaxime (ctx), and imipenem (imi) are shown.

	MLST	PMQR	Beta-lactamases	Other resistance genes	cip	lev	mox	del	caz	ctx	imi
*E. coli* **35**	ST131	*aac(6’)-Ib-cr*	*bla*_OXA−1_, *bla*_CTX−M−15_	*dfrA17*, *sul1*	128	2	16	8	4	128	0.06
*E. coli* **133**	ST15840	*qnrB19*	*bla*_CMY−2_	*dfrA1*,* sul1*,* sul2*	32	8	64	64	128	32	4
*E. coli* **149/1**	ST162	none	*bla*_CMY−2,_ *bla*_TEM−1B_	*dfrA17*	16	2	8	2	128	128	2
*E. coli* **260**	ST57	*qnrS1*	*bla*_TEM−1B_	*aac(3)-IId*	64	16	32	64	8	2	0.06
*E. coli* **269/1**	ST57	*qnrS1*	*bla*_TEM−1B_	*aac(3)-IId*	32	8	128	64	8	2	0.5
*E. coli* **706/1**	ST131	*aac(6’)-Ib-cr*	*bla*_OXA−1,_ *bla*_CTX−M−15_	*aac(3)-IIa*	128	8	32	8	128	128	1

## Discussion

Antibiotic resistant *E. coli* strains are a great challenge worldwide because limited number of effective antibiotics are available to treat infections caused by these resistant pathogens. However, several new agents from already known antibiotic groups have been approved for clinical applications in the past years, that can be used to treat multidrug-resistant bacterial infections. Among these new agents we can find ceftazidime-avibactam, ceftolozane-tazobactam, meropenem-vaborbactam, plazomicin as well as new fluoroquinolones^35^. Delafloxacin is a novel fluoroquinolone agent, that has an unique chemical structure and has more binding sites on the target molecules compared to earlier fluoroquinolones. Delafloxacin has been approved for clinical application however, resistance against this fluoroquinolone agent has been rarely reported, yet. In our earlier study, we analyzed prevalence of delafloxacin resistance in *E. coli* and we detected delafloxacin resistant *E. coli* ST43 in Hungary^34^.

In our current study, six delafloxacin resistant *E. coli* strains were investigated by WGS and according to genome sequence analysis multiple mutations in QRDRs were detected. Interestingly, three and five QRDR mutations were detected, that confer resistance to delafloxacin as well as to earlier fluoroquinolones (e.g.: ciprofloxacin, levofloxacin, moxifloxacin).

Taking into account that delafloxacin has more binding sites on the two target molecules, compared to previous fluoroquinolones, we hypothesize that different and more QRDR mutations are necessary to develop resistance againts this new agent. A recently published study revealed that *H. pylori* with *gyrA* Asn87Ile mutation was associated with dual levofloxacin and delafloxacin resistance, while *gyrA* Asn87Lys mutation maintain sensitivity to delafloxacin^36^.

Other bacterial species have different types of QRDR mutations that lead to delafloxacin resistance. In *S. aureus gyrA* with Ser84Leu, Glu88Lys, Ser84Leu, Ser85Pro alterations and *parC* with Ser80Tyr/Phe, Glu84Gly, Asp36Gly alterations lead to delafloxacin resistance^37^. GrlA with Ser80Phe and GrlB with Pro451Ser substitutions detected in methicillin-resistant *S. aureus* (MRSA) and a combination of at least three mutations in GrlA, GrlB, and/or GyrA is required to increase the MICs of fluoroquinolones^38^. Another study concluded that single point mutations at both *gyrA* and *grlA* in MRSA isolates resulted in resistance to ciprofloxacin, levofloxacin and moxifloxacin, but not to delafloxacin. Triple mutations in the QRDR were required to achieve resistance to delafloxacin amongst MRSA isolates, which strenghtens our current study with similar findings^39,40^. Similarly, in *N. gonorrhoeae* isolates, multiple *gyrA* and *parC* mutations results in delafloxacin resistance^30^.

Topoisomerase IV *parC* Glu84Val found in high-level delafloxacin-resistant isolates of both *E. coli* and *S. aureus* but not in those with MIC values < 1 mg/L. Previous studies have also found mutations in the same position suggesting that mutations in ParC Glu84Val could be responsible for delafloxacin resistance^34,41^.

Exploring the changes in target enzymes through mutations that were detected in our study, we have found the following results: *gyrA* mutation of Ser83Leu serine is a neutral, polar molecule substitution to hydrophobic amino acid leucine; Asp87Asn aspartate is a charged, acidic target molecule, which was substituted by a neutral amino acid asparagine. In *parC* Ser80Ile serine is a neutral, polar molecule substitution to hydrophobic isoleucine; Glu84Val glutamate is an acidic and charged amino acid compared to valine which is a hydrophobic molecule. All these substitutions lead to unfavourable changes to delafloxacin (and to other fluoroquinolenes) to bind to the target sites on enzymes. The more hydrophobic the target site becomes, the more increase in delafloxacin MIC is detected. However, in case of *parE* point mutation Ile549Leu, both isoleucine and leucine are hydrophobic, so we cannot assess the relevance of this mutation. We have not found any isolate which harbours only this single *parE* mutation, this specific mutation occured only in combination with other amino acid substitutions.

Additionally, PMQR determinants were also detected in five out of six delafloxacin resistant *E. coli*, that can enhance delafloxacin resistance, because higher delafloxacin MIC values (64 mg/L, Table 1) were detected in *E. coli* strains producing QnrS1 and QnrB19 determinants and having triple QRDR mutations, compared to *E. coli* strain lacking Qnr determinants but having triple mutations in QRDR (Table 1; Fig. 2). Qnr determinants are pentapeptid repeat proteins, that are capable to bind to gyrase and topisomerase enzymes in order to protect these enzymes from fluoroquinolone action^18^. Several Qnr determinants have been earlier reported in different strains of Enterobacterales in Hungary, and *qnrA*, *qnrB*, and *qnrS* were the most frequently detected in *E. coli* strains^42,43^.

Association between delafloxacin resistance and beta-lactamase production was also detected in our study namely, CTX-M-15 and OXA-1 in ST131; CMY-2 in ST162 and in ST15840; TEM-1B in ST57 and in ST162.

We identified a new sequence type namely, ST15840, that is closely related to ST69, and only a single allelic variation of *icd* indicates difference between these two STs. ST15840 is a member of clonal complex 69. *E. coli* ST69 is a well-known multidrug resistant clone, that has been reported worldwide from different clincal specimens as well as from environmental samples^3,4,44,45^. High number and different resistance determinants were already reported in *E. coli* ST69 namely, *bla*_KPC−2_, *bla*_NDM−1_^46^, *bla*_CMY−6_, *bla*_CTX−M−15,_*bla*_CTX−M−27,_*mcr-1*, *fosA3*^6,47,48^.

In our study we detected CMY-2 producing *E. coli* ST162 from a clinical isolate. Interestingly, *E. coli* ST162 has been recently reported from animal samples in Hungary^49,50^. This clone has been detected in duck and in rook cloacal and ceacal samples moreover, certain antibiotic resistance genes were reported in *E. coli* ST162 namely, *mcr-1*^43^ and *bla*_CTX−M−1_, *bla*_CTX−M−55_, *bla*_TEM−1B_, *qnrS1*^50^. All these results of *E. coli* ST162 in Hungary indicate importance of „One health” issue in antibiotic resistance, because antibiotic resistant *E. coli* can occur and circulate between different niches (e.g.: animal, environmental, human host) and antibiotic resistant *E. coli* strains can be selected out moreover, acquisition of different resistance genes and plasmids is enhanced^47,51,52^.

*E. coli* ST131 is a major high-risk clone and it is disseminated worldwide^3,53–55^. It has been reported as a multidrug-resistant clone capable of acquiring diverse resistance mechanisms^1,6^. ST131 was first detected in Hungary during a study in 2006–2007 and CTX-M-15 production was commonly seen in this clone^56^. Recently, further studies reported *E. coli* ST131 from clinical samples as well as from cloacal samples taken from rooks in Hungary, and interestingly, CTX-M-15 and CTX-M-27 producing strains were detected^50,57,58^. In our study, we analyzed clinical samples and two *E. coli* strains belonged to ST131 clone and both produced CTX-M-15 beta-lactamase.

Earlier studies demonstrated that multidrug-resistant *E. coli* high-risk clones possess double serin mutations in QRDR sequences. These are considered as beneficial mutations, because these can contribute to a favourable fitness cost of high-risk clones during development of fluoroquinolone resistance. Generally, fitness cost is associated with development of different resistance mechanisms in major bacterial pathogens however, a dissimilar fitness cost was described in high-risk clones of *E. coli* and *K. pneumoniae*. The favourable fitness cost enables high-risk clones to survive and to persist in human host or in the environment (e.g.: hospital environment) and to disseminate further^59,60^. In our study all six delafloxacin resistant *E. coli* strains possessed double serin mutaions in QRDR (Fig. 2), this also indicates, that delafloxacin resistance can commonly occur in high-risk *E. coli* clones.

## Conclusion

In this study we analyzed molecular mechanisms of delafloxacin resistance in *E. coli*. We conclude that, multiple mutations in coding genes of gyrase and topisomerase IV are required for delafloxacin resistance. Notably, three mutations in *gyrA* Ser83Leu, Asp87Asn, and *parC* Ser80Ile are capable to achive delafloxacin resistance in *E. coli*. We can also conclude that the presence of hydrophobic amino acids in biding sites of target enzymes play central role in delafloxacin reistance. In our study delafloxacin resistance was detected in different clones of *E. coli* namely, ST131, ST162, ST57 and ST15840. In this study we identified a new sequence type, ST15840 of CC69. We report CMY-2 producing *E. coli* ST162 from a clinical isolate in Hungary.

## Data Availability

Data availabilityAll six delafloxacin resistant E. coli whole genome sequences are submitted to NCBI at the following Bioproject PRJNA1115113, and Sequence Read Archive (SRA) accession numers E. coli 35 SAMN41510249; E. coli 133 SAMN41510250; E. coli 149/1 SAMN41510251; E. coli 260 SAMN41510252; E. coli 269 SAMN41510253; E. coli 706/1 SAMN41510254.Genomic data of E. coli ST15840 are submitted to Enterobase database at SE-LKH-E. coli 133 name.
